# Correction to “Novel long non‐coding RNA LINC02323 promotes epithelial‐mesenchymal transition and metastasis via sponging miR‐1343‐3p in lung adenocarcinoma”

**DOI:** 10.1111/1759-7714.14999

**Published:** 2023-06-12

Zhang X, Du L, Han J, Li X, Wang H, Zheng G, Wang Y, et al. Novel long non‐coding RNA LINC02323 promotes epithelial‐mesenchymal transition and metastasis via sponging miR‐1343‐3p in lung adenocarcinoma. Thorac Cancer. 2020;11(9):2506–16. https://doi.org/10.1111/1759-7714.13562.

The authors noticed an error in Figure 7C. The correct figure is shown below:

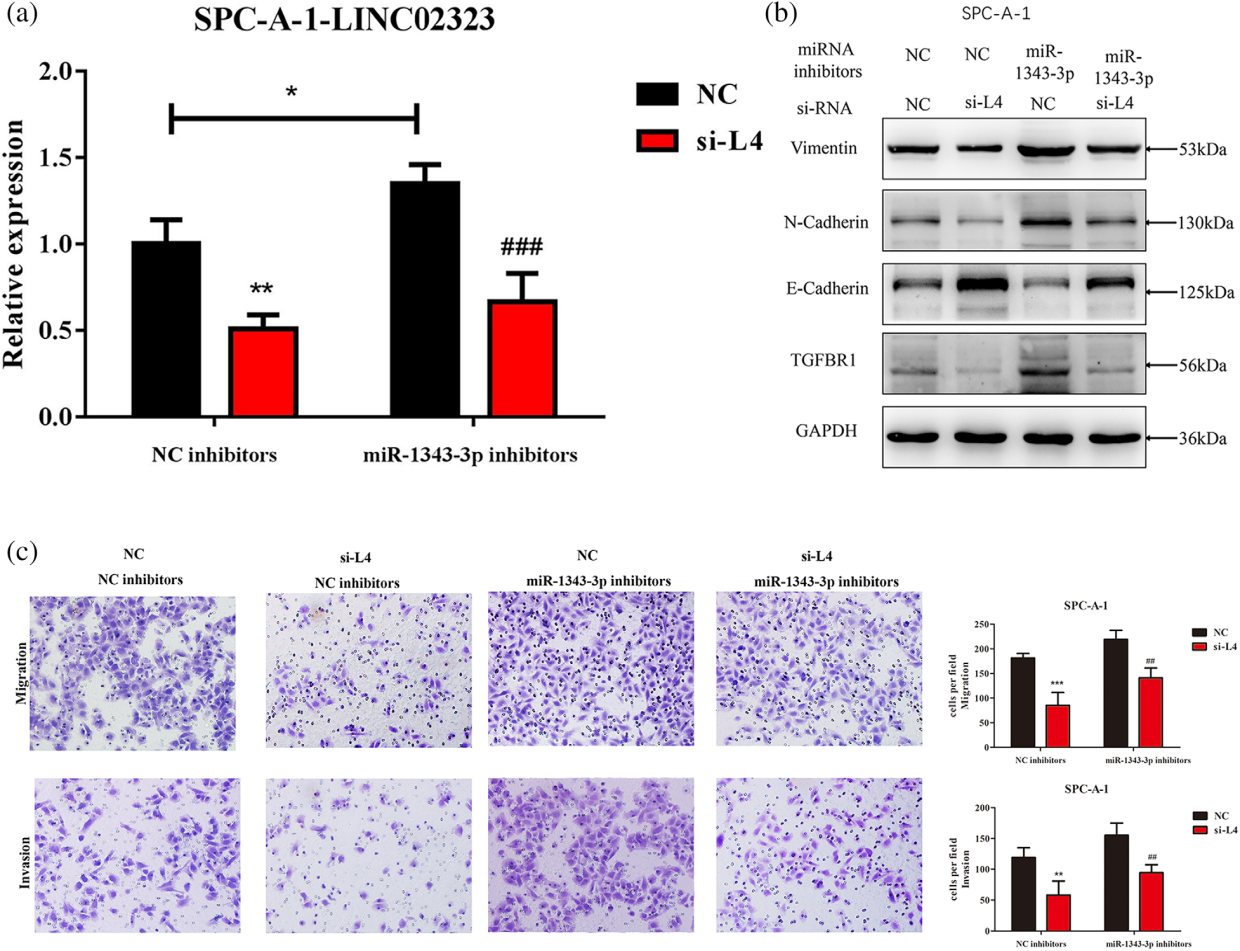

We apologize for this error.

